
JOURNAL INFORMATION
==============================
NLM Title Abbreviation: Orthop Surg
Journal ID: Orthop Surg
NLM Journal ID: 101501666
Journal ID: OS

Orthopaedic Surgery

ISSN: 1757-7853
EISSN: 1757-7861
Publisher: Wiley

ARTICLE INFORMATION
==============================
PMCID: PMC11474499
DOI: 10.1111/os.13782
Article ID: OS13782
Article version: 1
Subjects: Issue Information, Issue Information

Issue Information

Electronic publication date: 2024 Feb 1
Publication date: 2024 Feb
Volume: 16
Issue: 2
First page: 287
Last page: 288
Copyright: © 2024 The Authors. Orthopaedic Surgery published by Tianjin Hospital and John Wiley & Sons Australia, Ltd.
License: This is an open access article under the terms of the http://creativecommons.org/licenses/by/4.0/ License, which permits use, distribution and reproduction in any medium, provided the original work is properly cited.
License URL: https://creativecommons.org/licenses/by/4.0/

Figure count: 0
Table count: 0
Page count: 1

source-schema-version-number: 2.0
cover-date: February 2024
details-of-publishers-convertor: Converter:WILEY_ML3GV2_TO_JATSPMC version:6.4.9 mode:remove_FC converted:15.10.2024

==============================

Volume 16 • Issue 2 • February 2024 • ISSN 1757-7861

### Orthopaedic Surgery

Open Access

REVIEW ARTICLE

289 MODEL PROPERTIES AND CLINICAL APPLICATION IN THE FINITE ELEMENT ANALYSIS OF KNEE JOINT: A REVIEW

CLINICAL ARTICLES

303 THE ASSOCIATION BETWEEN HIGH PREOPERATIVE MRI-BASED VERTEBRAL BONE QUALITY (VBQ) SCORE AND TITANIUM MESH CAGE SUBSIDENCE AFTER ANTERIOR CERVICAL CORPECTOMY AND FUSION

312 COMPARISON OF BONE-SETTING ROBOTS AND CONVENTIONAL REDUCTION IN THE TREATMENT OF INTERTROCHANTERIC FRACTURE: A RETROSPECTIVE STUDY

320 EXPLORING CAUSAL RELATIONSHIPS BETWEEN LEUKOCYTE TELOMERE LENGTH, SEX HORMONE-BINDING GLOBULIN LEVELS, AND OSTEOPOROSIS USING UNIVARIABLE AND MULTIVARIABLE MENDELIAN RANDOMIZATION

329 COMPARISON OF CERVICAL SAGITTAL PARAMETERS AMONG PATIENTS WITH NECK PAIN AND PATIENTS WITH CERVICAL SPONDYLOTIC RADICULOPATHY AND CERVICAL SPONDYLOTIC MYELOPATHY

337 OUTCOMES OF RIGIDFIX CROSS PIN FIXATION IN FEMORAL AND TIBIAL TUNNEL FOR ANTERIOR CRUCIATE LIGAMENT RECONSTRUCTION

346 EFFECTS OF CERVICAL PARAVERTEBRAL EXTENSORS IN PATIENTS WITH CERVICAL OSSIFICATION OF THE POSTERIOR LONGITUDINAL LIGAMENT GROUPED ACCORDING TO mK-LINE

357 A RARE COMBINED INJURY IN CHILDREN DURING SIDE IMPACT: THE POSSIBLE MECHANISM AND TREATMENT RESULTS

363 NO SUPERIOR BONE UNION OUTCOMES WITH ALLOGRAFTS COMPARED TO NO GRAFTS AND AUTOGRAFTS FOLLOWING MEDIAL OPENING WEDGE HIGH TIBIAL OSTEOTOMY: A RETROSPECTIVE COHORT STUDY

374 INTERCALARY PROSTHETIC RECONSTRUCTION WITH THREE-DIMENSIONAL-PRINTED CUSTOM-MADE POROUS COMPONENT FOR DEFECTS OF LONG BONES WITH SHORT RESIDUAL BONE SEGMENTS AFTER TUMOR RESECTION

383 BLOOD PRESSURE FLUCTUATION PATTERN ASSOCIATED WITH 90-DAY ISCHEMIC STROKE AND TRANSIENT ISCHEMIC ATTACK AFTER TOTAL KNEE ARTHROPLASTY—A RETROSPECTIVE STUDY

391 THE NOMOGRAM MODEL AND FACTORS FOR THE POSTOPERATIVE MORTALITY OF ELDERLY PATIENTS WITH FEMORAL NECK FRACTURE UNDERGOING ARTIFICIAL HIP ARTHROPLASTY: A SINGLE-INSTITUTION 6-YEAR EXPERIENCE

401 ANALYSIS OF THE SCREW ACCURACY AND POSTOPERATIVE EFFICACY OF SCREW PLACEMENT IN SINGLE POSITION AND BIPEDAL POSITION IN ROBOT-ASSISTED OBLIQUE LUMBAR INTERBODY FUSION: PRELIMINARY RESULTS OF MAZOR X STEALTH USAGE

412 THE PRESERVED THICKNESS RATIO OF THE FEMORAL HEAD CONTRIBUTES TO THE COLLAPSE PREDICTOR OF OSTEONECROSIS

420 FORAMINOPLASTY PERFORMED WITH A TREPHINE AND A NEW TOOL IN TRANSFORAMINAL ENDOSCOPIC LUMBAR DISCECTOMY: A SINGLE-CENTER RETROSPECTIVE STUDY

429 HEALTH-RELATED QUALITY OF LIFE (HRQOL) OUTCOMES OF SELECTIVE/NONSELECTIVE THORACIC FUSION FOR LENKE 1C ADOLESCENT IDIOPATHIC SCOLIOSIS (AIS) PATIENTS WITH A MINIMUM 4-YEAR FOLLOW-UP

437 EPIDEMIOLOGY OF LATERAL PATELLAR DISLOCATION INCLUDING BONE BRUISE INCIDENCE: FIVE YEARS OF DATA FROM A TRAUMA CENTER

444 L4/5 DISC HERNIATION: NOT UNUSUALLY ACCOMPANIED WITH L5/S1 LOW-GRADE SPONDYLOLYTIC SPONDYLOLISTHESIS

RESEARCH ARTICLES

452 A RADIOGRAPHIC ANALYSIS OF CORONAL MORPHOLOGICAL PARAMETERS OF LOWER LIMBS IN CHINESE NON-KNEE OSTEOARTHRITIS POPULATIONS

462 EXPLORATION IN THE MECHANISM OF GINSENOSIDE Rg5 FOR THE TREATMENT OF OSTEOSARCOMA BY NETWORK PHARMACOLOGY AND MOLECULAR DOCKING

471 CHANGES IN THE LATERAL ACROMION ANGLE IN ROTATOR CUFF TEAR PATIENTS WITH ACROMIOPLASTY

481 A NOVEL PREDICTOR OF THE LENGTH AND SIZE OF ACL GRAFTS IN CHINESE HAN ADULTS FOR ACL RECONSTRUCTION: AN MRI STUDY

OPERATIVE TECHNIQUES

490 A NOVEL TECHNIQUE OF ARTHROSCOPIC-ASSISTED FOUR-CORNER FUSION AND ROBOT-ASSISTED FIXATION FOR SCAPHOID NONUNION ADVANCED COLLAPSE WRIST: A SINGLE CASE STUDY

497 HOW TO APPLY INTRAOPERATIVE ULTRASOUND WHEN SPINAL TRAUMA SURGERY IS PERFORMED IN THE LATERAL DECUBITUS POSITION?

CASE REPORTS

506 OSTEOCHONDRAL REGENERATION IN THE KNEE JOINT WITH AUTOLOGOUS PERIPHERAL BLOOD STEM CELLS PLUS HYALURONIC ACID AFTER ARTHROSCOPIC SUBCHONDRAL DRILLING: REPORT OF FIVE CASES

514 PERCUTANEOUS FULLY-ENDOSCOPIC ANTERIOR TRANSCORPOREAL PROCEDURE FOR THE TREATMENT OF ISOLATED OSSIFICATION OF THE POSTERIOR LONGITUDINAL LIGAMENT IN THE CERVICAL SPINE: A CASE REPORT

WILEY

CHINESE ORTHOPAEDIC ASSOCIATION
中华医学会骨科学分会

View this journal online at wileyonlinelibrary.com/journal/os

### Orthopaedic Surgery

**Honorary Editor-in-Chief**
Guixing Qiu, *Beijing, China*

**Editor-in-Chief**
Xinlong Ma, *Tianjin, China*

**Associate Editors**
Cyrus Cooper, *Southampton, UK*
Benjamin G. Domb, *Des Plaines, USA*
Qing Jiang, *Nanjing, China*
Baoge Liu, *Beijing, China*
Zhuojing Luo, *Xi'an, China*
Luigi Meccariello, *Moiano, Italy*
Ling Qin, *Hong Kong, China*
Qi Yin, *Liverpool, UK*

**Editorial Board**
Elisa Belluzzi, *Padua, Italy*
Carlo Biz, *Padua, Italy*
Mary L Bouxsein, *Boston, USA*
Richard E. Buckley, *Calgary, Canada*
Zhengdong Cai, *Shanghai, China*
Wei Chai, *Beijing, China*
Bohua Chen, *Qingdao, China*
Liang Chen, *Soochow, China*
Yanxi Chen, *Shanghai, China*
Pramod Devkota, *Patan, Nepal*
Meng Fan, *Tianjin, China*
Haoyu Feng, *Taiyuan, China*
Maoqi Gong, *Beijing, China*
Yong Hai, *Beijing, China*
Wei Huang, *Chongqing, China*
Baochao Ji, *Urumqi, China*
Ayhan Kanat, *Rize, Turkey*
Kenny Kwan, *Hong Kong, China*
Sallie Lamb, *Exeter, UK*
Lifeng Lao, *Shanghai, China*
Jung-Hee Lee, *Seoul, Korea*
Guanghua Lei, *Changsha, China*
Guoan Li, *Newton, USA*
Hui Li, *Tianjin, China*
Jianmin Li, *Jinan, China*
Weishi Li, *Beijing, China*
Jianhao Lin, *Beijing, China*
Fanxiao Liu, *Munich, Germany*
Ruben Lostado, *La Rioja, Spain*
Songchen Lyu, *Harbin, China*
Weihu Ma, *Ningbo, China*
Xin Ma, *Shanghai, China*
Masaya Nakamura, *Tokyo, Japan*
Muthu Sathish, *Velayuthampalayam, India*
Wolfgang Senker, *Linz, Austria*
Sancar Serbest, *Boston, USA*
Cailiang Shen, *Hefei, China*
Bin Shen, *Chengdu, China*
Huiyong Shen, *Shenzhen, China*
Jianxiong Shen, *Beijing, China*
Puyi Sheng, *Guangzhou, China*
Junying Sun, *Soochow, China*
Shui Sun, *Jinan, China*
YasuhikoTakegami, *Nagoya, Japan*
Sakae Tanaka, *Tokyo, Japan*
Xiaodong Tang, *Beijing, China*
Fei Wang, *Shijiazhuang, China*
Weijun Wang, *Nanjing, China*
Xu Wang, *Shanghai, China*
Zheng Wang, *Beijing, China*
Jidapa Wongcharoenwatana, *Bangkok, Thailand*
Chi-Chuan Wu, *Taipei, China*
Nan Wu, *Beijing, China*
Zhihong Wu, *Beijing, China*
Yayi Xia, *Lanzhou, China*
Jianru Xiao, *Shanghai, China*
Zhongyu Xie, *Shenzhen, China*
Dan Xing, *Beijing, China*
Weiguo Xu, *Tianjin, China*
Peng Xu, *Xi 'an, China*
CaoYang, *Wuhan, China*
Pei Yang, *Xi 'an, China*
Zhaoming Ye, *Hangzhou, China*
Xiuchun Yu, *Jinan, China*
Bin Yu, *Guangzhou, China*
Yi Zeng, *Chengdu, China*
Dianying Zhang, *Beijing, China*
Hong Zhang, *Texas, USA*
Weibin Zhang, *Shanghai, China*
Xianlong Zhang, *Shanghai, China*
Jianguo Zhang, *Beijing, China*
Bin Zhao, *Taiyuan, China*
Jie Zhao, *Shanghai, China*
Yu Zhao, *Beijing, China*
Zongke Zhou, *Chengdu, China*
Yue Zhu, *Shenyang, China*
Zezhang Zhu, *Nanjing, China*

**Address for Editorial Correspondence:** Editorial Board of *Orthopaedic Surgery*, Tianjin Hospital, 122 Munan Road, Tianjin 300050, China (email: orthopaedicsurgery@126.com; telephone: +86 22 2833 4734; fax: +86 22 2824 1184).

**Disclaimer:** The Publisher, Tianjin Hospital and Editors cannot be held responsible for errors or any consequences arising from the use of information contained in this journal; the views and opinions expressed do not necessarily reflect those of the Publisher, Tianjin Hospital and Editors, neither does the publication of advertisements constitute any endorsement by the Publisher, Tianjin Hospital and Editors of the products advertised.

**Copyright © 2024 The Authors. Orthopaedic Surgery published by Tianjin Hospital and John Wiley & Sons Australia, Ltd**

**Copyright and Copying (in any format)**
Copyright © 2024 The Authors. Orthopaedic Surgery published by Tianjin Hospital and John Wiley & Sons Australia, Ltd. All rights reserved. No part of this publication may be reproduced, stored or transmitted in any form or by any means without the prior permission in writing from the copyright holder. Authorization to copy items for internal and personal use is granted by the copyright holder for libraries and other users registered with their local Reproduction Rights Organisation (RRO), e.g. Copyright Clearance Center (CCC), 222 Rosewood Drive, Danvers, MA 01923, USA (www.copyright.com), provided the appropriate fee is paid directly to the RRO. This consent does not extend to other kinds of copying such as copying for general distribution, for advertising or promotional purposes, for republication, for creating new collective works or for resale. Permissions for such reuse can be obtained using the RightsLink "Request Permissions" link on Wiley Online Library. Special requests should be addressed to: permissions@wiley.com

For submission instructions, subscription and all other information visit: http://wileyonlinelibrary.com/journal/os

ISSN 1757-7861 (Online)

OS.JEB.February24
